# Impact of Heat and Pressure Processing Treatments on the Digestibility of Peanut, Hazelnut, Pistachio and Cashew Allergens

**DOI:** 10.3390/foods13223549

**Published:** 2024-11-07

**Authors:** Claudia Arribas, Africa Sanchiz, Mercedes M. Pedrosa, Selene Perez-Garcia, Rosario Linacero, Carmen Cuadrado

**Affiliations:** 1 Food Technology Department, Consejo Superior de Investigaciones Científicas-Instituto Nacional de Investigación y Tecnología Agraria y Alimentaria (CSIC-INIA), Ctra. La Coruña Km. 7.5, 28040 Madrid, Spain; arribas.claudia@inia.csic.es (C.A.); mmartin@inia.csic.es (M.M.P.); selene.perez@inia.csic.es (S.P.-G.); cuadrado@inia.csic.es (C.C.); 2 Genetics, Physiology and Microbiology Department, Biology Faculty, Complutense University, 28040 Madrid, Spain; charolin@ucm.es

**Keywords:** peanut, hazelnut, pistachio, cashew, food allergy, heat/pressure treatments, in vitro digestion, INFOGEST

## Abstract

Food processing can alter protein biochemical properties, impacting immunoreactivity and allergenicity. A key feature of food allergens is their resistance to enzymatic digestion, particularly by pepsin and trypsin. This study compares the digestomes of raw and heat- and/or pressure-treated peanuts, hazelnuts, pistachios and cashews using the INFOGEST harmonized digestion protocol and analyzing their IgE-binding capacity through in vitro methods. Protein patterns from controls and digestomes were resolved by SDS-PAGE and tested with sera from allergic patients, confirmed by competitive ELISA for hazelnuts and peanuts. The results indicate that processing methods differently affect the gastrointestinal (GI) digestion of these allergens. Simulated GI digestion caused a significant destruction of protein structures, reducing but not eliminating IgE reactivity for all four nuts. Boiling for 60 min did not change the SDS-PAGE profiles, but it did stimulate enzymatic activity, decreasing IgE binding capacity. In contrast, applying heat and pressure led to a nearly complete inhibition of allergenic potential during simulated digestion. These findings suggest that employing intense food processing techniques and investigating the gastrointestinal effects of highly allergenic nuts could be crucial steps toward developing new hypoallergenic formulations.

## 1. Introduction

Tree nuts and peanuts are, after fruits, the main predominant foods causing allergic reactions in Europe [1]. Over the last decades, there has been an increase in nut and tree nut consumption because of their favorable health benefits and singular nutritional composition, including vitamins, amino acids and phenolic acids [2,3]. Vicilins, legumins, 2S albumins, nsLTPs (non-specific lipid transfer proteins), profilins and pathogenesis-related (PR) proteins are the main protein families causing tree nut allergy. Moreover, thaumatin-like and oleosin are relevant protein allergens [4]. Tree nut allergens are characterized by a high resistance to denaturation and enzymatic degradation. Currently, at least 11 allergens have been identified in peanut: Ara h 1 (7S vicilin), Ara h 3 and Ara h 4 (11S legumins), Ara h 5 (profilin), Ara h 2, Ara h 6 and Ara h 7 (2S albumins), Ara h 8 (PR-10), Ara h 9 (LTP, lipid transfer proteins), Ara h 10 and Ara h 11 (oleosins) [5]. Ara h 1 constituted around 15% of total proteins, while Ara h 2 constituted about 8%. Several hazelnut proteins have been identified and included on the WHO-IUS list of allergens and in the Allergome database [6,7,8]: Cor a 1 (Bet v1 homologue), Cor a 2 (profilin), Cor a 8 (LTP), Cor a 9 (11S legumin), Cor a 11 (7S vicilin), Cor a 14 (2S albumin) and Cor a 12, Cor a 13 and Cor a 15 (oleosins) [9]. The five major allergens of pistachio are Pis v 1 (2S albumin), two 11S legumins (Pis v 2 and 5), the 7S vicilin Pis v 3 and the superoxide dismutase Pis v 4 [10,11,12]. In the case of cashew, three allergens have been identified so far: Ana o 1 (7S vicilin), Ana o 2 (11S legumin) and Ana o 3 (2S albumin), which can induce severe reactions even at minimal doses [13,14]. Most epitopic regions of Pis v 1 and Pis v 3 are homologs with the epitopes of cashew allergens. This finding is considered the molecular base for the reported cross-reactivity between cashew and pistachio [15,16,17].

Food processing at the industrial level is useful to ensure food safety and improve organoleptic characteristics. Such food treatment could modify allergenicity because it could modify the properties of protein allergens. The type of processing used, conditions, food matrix, duration, etc., influence the changes that food proteins undergo during processing, including denaturation, aggregation and biochemical alterations [18,19]. Food proteins can undergo various changes during processing, including aggregation, denaturation and chemical modifications. These changes can affect IgE reactivity, potentially increasing or decreasing food allergenicity. Therefore, it is important to understand how food processing influences protein characteristics—such as their resistance to heat and pressure, as well as their chemical and mechanical properties—especially in the context of managing food allergies [20,21,22,23]. Thermal treatments can be boiling, roasting, frying and pressured heating by an autoclave or DIC (controlled instantaneous depressurization) processing. The non-thermal processing on foods, such as enzymatic digestion or HHP (high hydrostatic pressure), could be applied individually or in combinations. These processes could modify the physicochemical characteristics of proteins or produce biochemical reactions in the food matrix components [18,24]. The modifications of tree nut and peanut allergenicity after thermal processing have been extensively studied. Upon boiling (100 °C), the allergenicity of cashew, pistachio, almond and walnut is not affected, as well as for peanuts and hazelnuts [25,26,27,28]. However, a significant reduction in hazelnut allergenicity is achieved after roasting (140 °C for 40 min), although 29% of allergic patients still suffer clinical symptoms after the consumption of roasted hazelnuts. The autoclave effect on allergenicity of tree nuts has been investigated on hazelnut, walnut, almond, chestnut, cashew and pistachio, besides peanut [29,30,31,32]. These studies demonstrated a clear reduction in allergenicity after autoclaving these tree nuts, which was directly related with the heat/pressure and time increment. The final efficacy of autoclaving on almond and hazelnut immunoreactivity is enhanced through the application of the prehydration step (2 h) followed by drying treatment [27,28]. In addition, the digestibility of hazelnut allergens is higher in prehydrated samples than in the controls and at a higher protein fragmentation level. The influence of other pressured heat treatment, such as controlled instantaneous depressurization (DIC), has been analyzed for peanut and tree nut IgE reactivity [30,33,34]. Consistent with these findings, applying DIC under harsh conditions (7 bar, 120 s) led to a significant decrease in the IgE immunoreactivity of tree nut and peanut allergenic proteins. In vitro tests using IgE sera from sensitized patients (immunoblots and ELISA) showed that the reduction in immunodetection was more pronounced for pistachio (75%) compared to cashew, although it was not eliminated. This suggests that pistachio allergens are less resistant than cashew proteins. The combination of pressure-heating (autoclave and DIC) with enzymatic hydrolysis using food-grade proteases significantly lowered allergenic reactivity. Notably, the use of DIC treatment prior to enzymatic digestion proved to be the most effective approach for drastically reducing or even eliminating the allergenic potential of peanuts and tree nuts [30,31].

The resistance of proteins to gastrointestinal (GI) digestion represents an important factor for allergenicity evaluation. Higher resistance to digestion seems to increase the sensitization capacity of a food protein, and consequently, allergenicity is also incremented [35]. Stability under gastric conditions has been considered a useful parameter for the identification of allergens [36,37], and in vitro tests of pepsin digestion have been incorporated since 2001 into a FAO/WHO procedure for the allergenicity assessment of novel food proteins [33]. An allergen must preserve its intact structure during the digestion process to sensitize an individual via the oral route, thus allowing for the intact epitopes to be taken up by the gut to sensitize the mucosal immune system. However, potent allergens are not stable upon digestion, and the localization of multiple IgE-binding epitopes in structural elements that are not surface-exposed suggests that the partial digestion of food allergens in the GI tract does not eliminate their sensitizing capacities [32]. Previous studies have demonstrated that the in vitro digestion products of peanut allergens are able to induce IgE production in rats [38]. In vitro digestion models simulating the human digestion process are a useful approach to address this issue. The main advantages of this tool are simplicity, low costs and good reproducibility [39].

Thermal treatment combined or not with pressure (boiling for 60 min and autoclave at 256 kPa for 30 min) or without temperature (controlled instantaneous depressurization, DIC, at 7 bars for 2 min) has been applied because of its potential to reduce the immunoreactivity of peanut, hazelnut, cashew and pistachio nuts. We investigated the stability and IgE-binding capacity of the main allergenic proteins of these raw and treated nuts after applying gastrointestinal digestion conditions. The influence of GI digestion on main protein allergens was assayed for the first time in treated nuts, which is especially novel in the case of pistachio and cashew.

## 2. Materials and Methods

### 2.1. Plant Material

Peanuts (*Arachis hypogea*, variety Virginia) and commercial unprocessed cashews (*Anacardium occidentale* L. type 320) were supplied by Productos Manzanares (Spain). Hazelnut (*Corylus avellana*, var. Negreta) and pistachio nuts (*Pistacia vera* L. var. Kerman) were obtained from the IRTA (Institut de Recerca I Tecnologia Agroalimentaries; Mas de Bover, Tarragona, Spain). The nuts were collected in 2018, in the appropriate state of ripeness. All nuts were processed following Cuadrado et al. [25] to obtain seeds that were untreated, boiled at 100 °C for 60 min (in distilled water 1:5 *w*/*v*), autoclaved at 138 °C, 256 kPa for 30 min (in a food-grade autoclave, Compact 40 Benchtop Autoclave, Priorclave, London, UK) and treated with controlled instantaneous depressurization (DIC, 7 bars for 2 min). This last treatment was performed following a factorial experimental design by La Rochelle University (LaSiE) and described by Haddad et al. [40].

The processed seeds were homogenized and milled to a <1 mm particle size using a kitchen robot (Thermomix 31-1, Vorwerk Elektrowerke, GmbH & Co. KG, Wüppertal, Germany). Flours were defatted with n-hexane (34 mL/g of flour, 4 h with stirring) and air-dried, and the nitrogen content was determined by LECO analysis according to the Official Methods of Analysis [41]. The protein content was calculated with a conversion factor of 5.33 [37]. All treated and untreated flours were stored at 4 °C in polyethylene bags until analysis, such as the control samples (CS).

### 2.2. Enzymes and Reagents

α-amylase from porcine pancreas (A3176-500 KU; Type VI-B, lyophilized powder 5 U/mg solid), bile from bovine and ovine bile acid mixtures (B8381-25G), pancreatin from porcine pancreas (P7545-25G, lyophilized powder, 8 × USP) and porcine pepsin from gastric mucosa (P6887-5G, lyophilized powder 3200–4500 U/mg protein) were purchased from Sigma–Aldrich (Saint-Louis, MO, USA). All reagents used in the assay were standard analytical-grade.

### 2.3. Patient Sera

Sera from 32 patients allergic to peanut, hazelnut, cashew and pistachio were collected at the Allergy Department of any of the three Spanish hospitals (Hospital Universitario Cruces, Fundación Jiménez Díaz, and Hospital Universitario Princesa) during 2020–2022. The investigation was authorized by the Ethics Committees of the three hospitals, in agreement with the regulations of the boards of their organizations (permissions CBVI839/2M, PIC164-18, no. 3798, respectively).

Immunoreactivity against peanut and hazelnut was assayed using two different pools of sera. One of them included 10 sera from individuals sensitized to 2S albumin, 7S vicilin and/or 11S globulins peanut allergens, named Sprot (storage proteins) (patient nos. #3, #4, #5, #8, #9, #15, #17, #21, #27 and #31, all used at a 1:10 dilution). The other serum pool was formed by 17 peanut individuals sensitized to LTP allergen (#2, #6, #10, #11, #12, #13, #14, #16, #18, #19, #20, #25, #26, #28, #30, #32 and #33, all used at a 1:10 dilution). On the other hand, a pool of sera from 7 (#1, #3, #4, #5, #8, #22 and #27) or 15 patients (#2, #6, #10, #11, #13, #14, #16, #18, #19, #20, #23, #24, #26, #30 and #33) sensitized to hazelnut Sprot or LTPs, respectively, was prepared. The serum pool from patients with sensitization to pistachio and cashew (positive skin prick test and specific IgE > 0.35 to these nuts) was prepared with 15 sera (# 1, #2, #3, #5, #6, #8, #10, #14, #18, #23, #25, #27, #29, #31 and #33) and 12 sera (#1, #3, #4, #5, #6, #8, #10, #18, #25, #27, #31 and #33), respectively. Specific information about the included patients is listed in Table S1.

Total IgE and specific serum IgE levels were measured by ImmunoCAP^®^ (ThermoFisher Scientific, Upsala, Sweden). IgEs specific to peanut allergens (Ara h 1, Ara h 2, Ara h 3, Ara h 6, Ara h 8 and Ara h 9), hazelnut allergens (Cor a 1, Cor a 8, Cor a 9 and Cor a 14) and cashew allergens (Ana o 3) were also determined and collected following the manufacturer’s recommendations, taking into account that an sIgE value at least above 0.35 kUA/L is assumed as a positive result.

### 2.4. In Vitro Enzymatic Digestion

**Figure 1:**
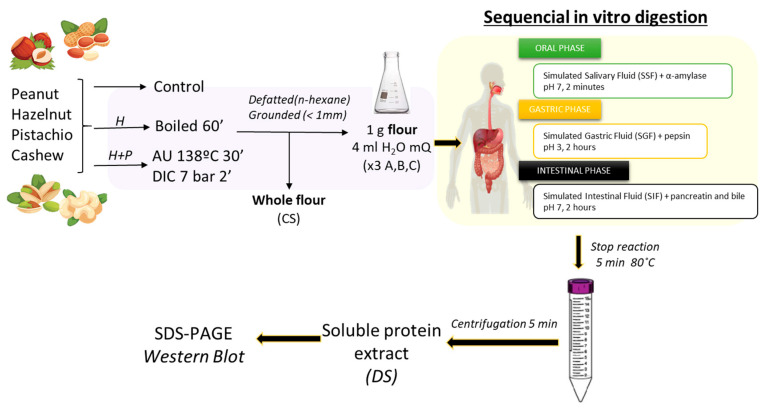
Experimental procedure for the in vitro static digestion model and analysis performed for peanut, hazelnut, pistachio and cashew allergens. *H*—heat; *P*—pressure; CS—control sample; DS—digested sample.

The in vitro digestion was sequentially performed following the INFOGEST protocol with slight modifications (Figure 1) [35]. This method simulated different conditions of the bolus along the gastrointestinal tract. All samples were performed in triplicate, A, B and C, obtaining the digested sample (DS). Appropriate controls without enzymes were also included (CS), being the nut whole extract from defatted flours (Figure 1).

To simulate the oral phase, 1 g of defatted flour of each sample was homogenized with MilliQ H_2_O, ratio 1:4 (*w*/*v*), and transferred to a flask. Another 1 mL of MilliQ H_2_O was added and shaken for 15 min at room temperature. At 37 °C under agitation, the sample was mixed with 0.7 mL of simulated salivary fluid (SSF) and 5 µL of CaCl_2_ 0.3 M. Subsequently, 195 µL of H_2_O MiliQ and 0.1 mL of α-amylase were added and incubated for 2 min at 37 °C under agitation (160 rpm).

To simulate the gastric phase, at room temperature, 1.5 mL of simulated gastric fluid (SGF) stock solution was added to the samples, and then 1 µL of CaCl_2_ 0.3 M. The pH was adjusted to pH 3 with HCl 3 N; finally, H_2_O MilliQ was added to complete the final volume of 2 mL. The flask was incubated for 1 h at 37 °C and 160 rpm orbital agitation, and 0.16 mL of pepsin was added. After this period, the samples were cooled to room temperature.

To simulate the intestinal phase, the samples were mixed with 2.2 mL of simulated intestinal fluid (SIF) and 8 µL CaCl_2_ 0.3 M. The pH was adjusted to pH 7 with NaOH 4 N and H_2_O MilliQ was added to the flask. Pancreatin (1 mL) and 0.5 mL of bile salts were mixed and incubated in the flask for 2 h at 37 °C and 160 rpm in orbital agitation (final volume 4 mL). Finally, the reaction was stopped at 80 °C for 5 min. After centrifugation for 5 min at 10,000× *g* at 4 °C, the supernatant was collected and frozen for subsequent analysis.

### 2.5. SDS-PAGE Analysis

Peanut samples (20 µg protein per well) were performed on 4–20% gradient polyacrylamide gels, according to the Laemmli method, using β-mercaptoethanol (Bio-Rad, Hercules, CA, USA) [42], and hazelnut, pistachio and cashew samples (20 µg protein per well) were loaded in 4–12% using the Bolt LDS sample buffer (Invitrogen, Carlsbad, CA, USA). The samples were boiled at 60 °C for 10 min and loaded in Tris-HCl linear gradient gels (Bio-Rad, CA, USA) and Bolt Bis-Tris Plus gels, respectively. Both protein gels were stained with the Coomassie Brilliant Blue dye. The molecular weight markers were Precision Plus (from 10 to 250 kDa, Bio-Rad) and See Blue (from 3 to 198 kDa, Invitrogen). The molecular weight of the bands was assessed using Quantity One software, version 4.6.1 (Bio-Rad, CA, USA).

### 2.6. Immunoblotting Western Blot Experiments

The samples (20 µg protein per well) were loaded and resolved on a 4–20% (Bio-Rad, CA, USA) or 4–12% (Invitrogen, Carlsbad, CA, USA) gel such as SDS-PAGE Analysis. Proteins were transferred onto PVDF membranes using transfer equipment (iBlot 2 Western Blot Transfer System, Invitrogen, Carlsbad, CA, USA) for 7 min at 20 volts. After transference, membranes were washed and incubated in a blocking solution (2% non-fat milk in PBS, at room temperature) for 1 h and then with a 1:10-diluted serum pool from patients at 4 °C overnight with agitation. Then, the membranes were washed with PBS 0.05% Tween20 and treated with horseradish peroxidase (HRP)-conjugated mouse anti-human IgE (Sigma, Saint Louis, MO, USA) diluted in a blocking solution (1:10,000). Finally, detection was performed with BCIP/NBT substrate (Sigma, Saint Louis, MO, USA) and the results revealed using the CCD camera system of the ChemiDoc (Bio-Rad, Hercules, CA, USA).

### 2.7. Inhibition ELISA

A competitive ELISA inhibition assay was conducted on peanut and hazelnut samples with some modifications [30]. The wells of polystyrene microtiter plates (Immulon 4 HBX, Thermo Scientific, Waltham, MA, USA) were coated with 100 μL of raw nut protein extracts (0.5 mg/mL in PBS, pH 7) and incubated overnight at 4 °C. Serum pools were prepared from allergic patients sensitized to Sprot, with the peanut pool comprising sera from nine patients (numbers #3, #4, #5, #8, #15, #17, #21, #27 and #31, diluted at 1:10) and the hazelnut pool from seven patients (numbers #1, #3, #4, #5, #8, #22 and #27, diluted at 1:10). These serum pools were preincubated overnight at 4 °C with shaking, in the presence of digested and undigested samples of raw (control), boiled, autoclaved and DIC-treated nuts (final concentrations: 0.1, 0.01 and 0.001 mg/mL). An uninhibited serum control (preincubated with PBS) was also included. Afterward, the wells were washed with PBS containing 0.1% Tween-20 and blocked with 100 μL of 3% non-fat milk in PBS for 1 h at 37 °C, followed by another wash. The uninhibited and inhibited sera (100 μL each) were added to the wells for 1 h. After washing, the samples were incubated with 100 μL of horseradish peroxidase (HRP)-conjugated mouse anti-human IgE (1:1000 dilution) (Sigma, Saint Louis, MO, USA) for 1 h at 37 °C and washed again. Following this, 100 μL of peroxidase substrate (SureBlue TM, KPL, Gaithersburg, MD, USA) was added for the peroxidase reaction (30 min). The reaction was stopped by adding 100 μL of 1% HCl. The optical density (O.D.) was measured at 450 nm, and the percentage of inhibition in IgE binding was calculated using the following formula:

**[(1 − (AI/AN)]** × **100**

where AI is the O.D. value of the samples preincubated with inhibited sera (raw or thermally or enzymatically digested samples) and AN is the O.D. value of the samples incubated with uninhibited sera. All tests were performed at least in triplicate.

### 2.8. Statistical Analysis

To compare the inhibition data from ELISA, a simple variance analysis (ANOVA) was carried out (3 replicates). Subsequently, a comparison of the means was carried out using the Duncan test and significant differences were considered when *p* < 0.05. All analyses were performed using the StatGraphic Centurion, version XVII program (Statpoint Tech. Inc., Warrenton, VA, USA).

## 3. Results and Discussion

### 3.1. General Characteristics of the Collected Sera

Thirty-two allergic patients were studied in this research (56.26% female, average age and standard deviation of 27.6 ± 14.74 years, ranging between 55 and 6 years), showing immunoreactivity to peanut, hazelnut, cashew and/or pistachio allergens. The total sIgE average was 1078.2 kU/L ± 293.4 (ranged between 7600 and 49 kU/L).

Sera were classified according to sensitivity to specific allergens (when the information was known) (Table S1). Thus, for peanut and hazelnut, two different sera pools were prepared for each nut, containing sera from patients sensitized to storage proteins from both nuts (named Sprot, being 2S albumins, 7S vicilins and 11S globulins, with 10 or 7 sera per nut, respectively) or to the LTP allergen (pools containing 16 and 15 sera, respectively). In the case of cashew and pistachio, for which less information was available about specific allergens, two pools were also prepared. One of them was a pistachio-sensitized pool, containing sera from 15 patients, and another for cashew-sensitized patients that contained 12 sera. Allergy to cashew and pistachio is frequently shared by the same patients. We found that 92% (11/12) of the sera containing sIgE to cashew were also sensitive to pistachio nuts, whereas 73% (11/15) of the patients with sIgE to pistachio (>0.35 kU/L) also showed sIgE to cashew. Interestingly, among the collected sera, none of the patients were allergic only to cashew and only one showed allergy only to pistachio (patient #29). Half of the patients allergic to cashew showed sIgE > 0.35 kU/L to the 2S albumin Ana o 3 (6/12). Most of the patients (81%) in this study were allergic to peanuts (26/32, 7 of them showed allergy only to this nut, 27%), followed by hazelnut (22/32, nearly 69%; two of them were allergic only to hazelnut, 9%). Almost 22% (7/32) of the patients were allergic to the four nuts assayed in this study (Figure S1A).

As mentioned before, specific IgE for common allergenic proteins (2S, 7S, 11S, PR-10 and LTP) were available for analysis, mostly from peanut and hazelnut (Table S1). All patients, except for one (#29), were allergic to one or both nuts; 19 of them were sensitized to LTP allergens (61%) and 12 to Sprot allergens (38.7%) (Figure S1B).

Among the reported symptoms, 50% of the patients showed oral allergy syndrome (OAS) (16/32), whereas almost 41% experienced systemic symptoms such as angioedema, vomiting or urticaria. Five out of 32 did not report or experience any allergenic symptoms, or it was not known (15.6%). None of them suffered anaphylaxis after the ingestion of these nuts (Table S1).

### 3.2. Influence of In Vitro Digestion on Untreated and Processed Samples

Food allergenicity is related to the usually high resistance of the protein allergen to digestive enzymes, and tree nuts are not an exception [43,44]. Commercially available enzymes are normally designed for improving food digestibility, some sensory properties or enhancing biological activities (such as anti-inflammatory, anti-microbial or antioxidant properties), and even reducing the presence of allergenic compounds [45]. Thus, controlled enzymatic digestion using specific proteases has been assayed as a processing tool to reduce the allergenicity of many tree nut allergens, including peanut, cashew, pistachio or hazelnut [25,34,46]. A key point in understanding the resistance of any allergenic protein is to know its susceptibility to GI digestion, so we aimed to examine the outcome of the in vitro gastric digestion of hazelnut, peanut, pistachio and cashew proteins by following the INFOGEST protocol [35]. Thus, the structural changes derived from thermal treatments of protein allergens might have a direct consequence on their GI digestibility, and a combination of both processes has been proved to be crucial for understanding and verifying the immunoreactivity of these nuts [47]. We investigated the effect of several thermal processing methods, combined with or not with pressure, in the GI digestibility of these allergenic proteins. We assayed, via the INFOGEST protocol, the complete flours of these tree nuts after defatting, since the presence of lipids, sugars and other molecules is a limiting factor that increases the complexity of the assay. In this work, we analyzed the effects of sequential in vitro digestion simulating oral, gastric and intestinal phases on the IgE-binding capacity of the mentioned tree nuts by Western blotting and ELISA.

#### 3.2.1. Effect of Processing on the Peanut Protein Profile and IgE-Binding Capacity After In Vitro Gastrointestinal Digestion

**Figure 2:**
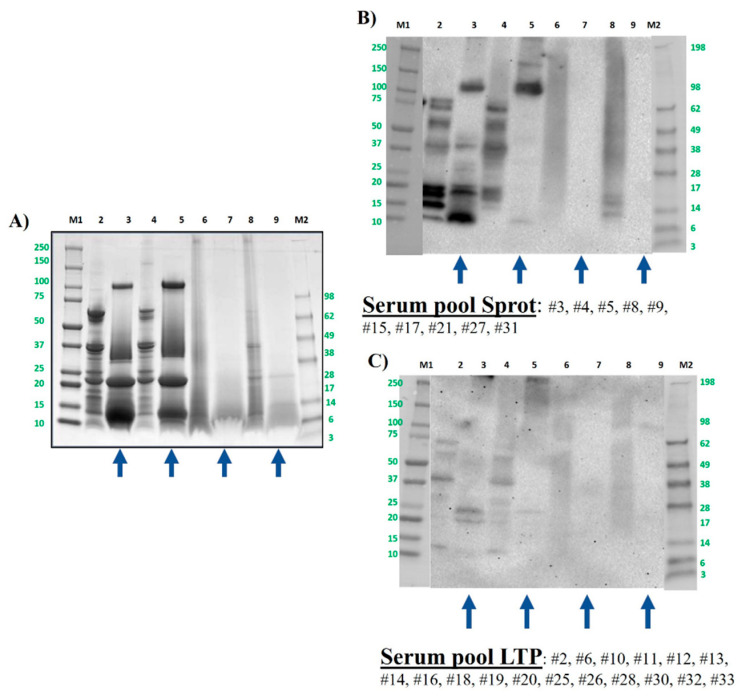
Peanut protein samples were analyzed by SDS-PAGE electrophoresis in 4–20% tris-glycine protein gels (**A**) and by immunoblotting using a pool of sera from peanut-allergic patients (grouped as 2S albumin- (**B**) or LTP-allergic individuals (**C**)). Lane 2: control, raw whole peanut flour (CS). Lane 3: control digested peanut (DS). Lane 4: boiled for 60 min whole peanut flour. Lane 5: boiled for 60 min digested peanut DS. Lane 6: AU 138 °C, 30 min whole peanut flour. Lane 7: AU 138 °C 30 min digested peanut DS. Lane 8: DIC 7b, 2 min whole peanut flour. Lane 9: DIC 7 b, 2 min digested peanut DS. Lanes M1 and M2: molecular weight marker Precision Plus and See Blue (10–250 kDa, BioRad and 3–198 kDa, Invitrogen, respectively). Laemmli loading buffer containing 4% of β-mercaptoethanol was used in all samples. Digested samples are highlighted with blue arrows under the figure.

Figure 2A shows the protein profile of peanut, including untreated and thermally treated samples by boiling or autoclaving under different conditions, before and after the INFOGEST assay. The GI digestion of raw peanut altered but did not reduce the protein profile and modified the IgE-binding peptides when it was assayed with both serum pools (Figure 2B, C). It can be noticed that boiling for 60 min has no effect on the protein band profile of peanut, showing the same band range from 60 to 10 kDa (Figure 2A, lane 4) approximately, being consistent with the previous results [44,48,49]. After the INFOGEST digestion of untreated and boiled samples, the peanut protein profile remained the same, but included a not-described protein or a protein accumulation of around 100 kDa (Figure 2A, lanes 2–5).

When Western blotting was analyzed, it could be observed that the in vitro digestion of boiled peanut reduces the IgE capacity of the serum pool from allergic patients, both in the Sprot pool and LTP pool. A digestion-resistant band was visible around 10 kDa in the assay with the Sprot pool (Ara h 2) [50]. Moreover, the protein of 100 kDa which did not correspond to any described allergen in peanut remained intact and with important IgE-binding capacity (Figure 2B, lanes 3 and 5). In the LTP pool, the Ara h 2 doublet bands between 20 and 25 kDa were affected by boiling, being only the heavier protein able to retain the binding of IgE in these samples. The band at around 10–12 kDa in this LTP pool, which corresponds to nsLTP allergens Ara h 9 or Ara h 16, was slightly reactive in raw and boiled samples, but it disappeared after GI digestion simulation (Figure 2C, lanes 3 and 5).

It has been already demonstrated that autoclaving, especially under drastic conditions such as the assay in this work (138 °C, 2.56 b and 30 min), reduces the capacity of IgE binding significantly [25]. The protein smear obtained after the in vitro digestion of this pressure-treated sample was almost complete, and the capacity of IgE binding was nearly inexistant (Figure 2B,C lane 7). Slightly less protein degradation extent can be observed in peanut samples treated by the DIC process, although an important elimination of protein bands was recovered after the INFOGEST digestion process (Figure 2A, lanes 8 and 9). Among the resistant protein bands, two bands are noticeable at about 15 and 12 kDa, which were still able to trigger IgE binding in the Sprot pool of sera. In the pool LTP, by contrast, the IgE-binding profile was practically the same before or after the INFOGEST of samples treated by autoclaving and DIC.

Recently, it was demonstrated that peanut pastes (instead of soluble extracts) required not only DIC treatment at specific conditions of 7 b for 2 min, but also enzymatic hydrolysis using commercial protease to complete the mitigation of IgE reactivity against storage-protein allergens [25].

#### 3.2.2. Effect of Processing on the Hazelnut Protein Profile and IgE-Binding Capacity After In Vitro Gastrointestinal Digestion

**Figure 3:**
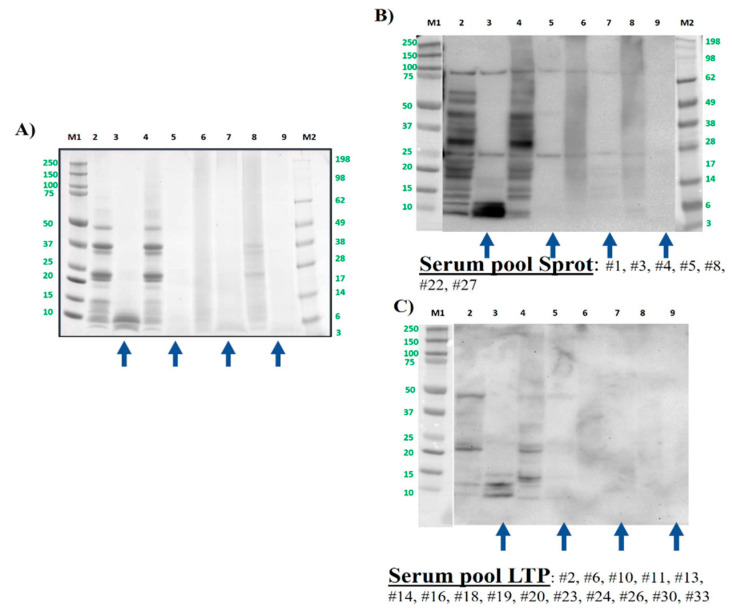
Hazelnut protein samples were analyzed by SDS-PAGE electrophoresis in MES 4–12% protein gels (**A**) and by immunoblotting using a pool of sera from hazelnut-allergic patients (distributed as 2S albumin- (**B**) or LTP-allergic individuals (**C**)). Lane 2: control, raw whole hazelnut flour (CS). Lane 3: control digested hazelnut (DS). Lane 4: boiled for 60 min whole hazelnut flour. Lane 5: boiled for 60 min digested hazelnut DS. Lane 6: AU 138 °C, 30 min whole hazelnut flour. Lane 7: AU 138 °C 30 min digested hazelnut DS. Lane 8: DIC 7b, 2 min whole hazelnut flour. Lane 9: DIC 7 b, 2 min digested hazelnut DS. Lanes M1 and M2: molecular weight markers Precision Plus and See Blue (10–250 kDa, BioRad and 3–198kDa, Invitrogen, respectively). Bolt LDS sample buffer (Invitrogen) + sample-reducing agent were used in all samples. Digested samples are highlighted with blue arrows under the figure.

The protein band pattern of treated and untreated hazelnut samples is shown in Figure 3A. The raw hazelnut protein profile ranged from 50 to 5 kDa approximately (lane 2). Although not visible in the SDS-PAGE gel, it was noticeable again a band of around 80 kDa in the immunoblotting using the Sprot pool of sera, especially in untreated and boiled samples, before and after digestion (Figure 3B, lanes 2–5). Apart from these bands, not recognized as a described allergen so far, it could be observed that the protein profiles of untreated and boiled samples were very similar, as well as the IgE-binding profile. The gastrointestinal digestion of untreated hazelnut decreased the immunoreactivity of most of the allergenic bands, but others seemed to suffer an increase in the IgE-binding capacity. The in vitro digestion of boiled samples with the simulated GI protocol showed a slightly higher extent than in raw samples, and low-molecular-weight allergenic protein was lost when both serum pools were assayed (Figure 3B,C, lanes 3 vs. 5).

Thermal treatments, such as boiling, modify the structure of allergens, making them more accessible to enzymes pepsin, trypsin or chemo trypsin [51]. In the Sprot pool, the intense immunogenic band at around 10 kDa corresponds to Cor a 14, the only characterized 2S albumin from hazelnut. In fact, the small and large subunits described for this allergen that are also known to be highly resistant proteins to temperature and GI digestion could be distinguished. Moreover, a discrete band in the 25 kDa area, probably corresponding to the basic subunit of Cor a 9, also remained undigested, but the acidic subunit, expected at around 40 kDa, was not visible. Finally, the intense immunoreactive band in the range of 30 kDa observed in untreated and boiled samples (Figure 3B, lanes 2 and 4) disappeared in the digested samples, probably converted to shorter peptides. Regarding the serum pool called the LTP pool, we observed that the GI digestion of untreated hazelnut, which showed a quite complex profile of multiple immunogenic protein bands affected the IgE-binding capacity, importantly, except for the doublet at around 6–12 kDa corresponding to the LTP allergen named Cor a 8, even more visible after in vitro digestion (Figure 3B, lane 3). All these results are in concordance with those obtained recently by other authors analyzing the impact of the INFOGEST digestion of roasted and control hazelnuts [52].

The treatments autoclave and DIC were almost unable to trigger a reaction with IgE in the serum, independent of the assayed pool, even before performing the in vitro digestion. The extraction of proteins during digestion is crucial in these in vitro protocols, particularly when thermal treatments are involved; such treatments can cause cross-linking and unexpected reactions that promote protein oligomerization, thereby reducing the efficiency of protein extraction.

#### 3.2.3. Effect of Processing on the Pistachio Protein Profile and IgE-Binding Capacity After In Vitro Gastrointestinal Digestion

**Figure 4:**
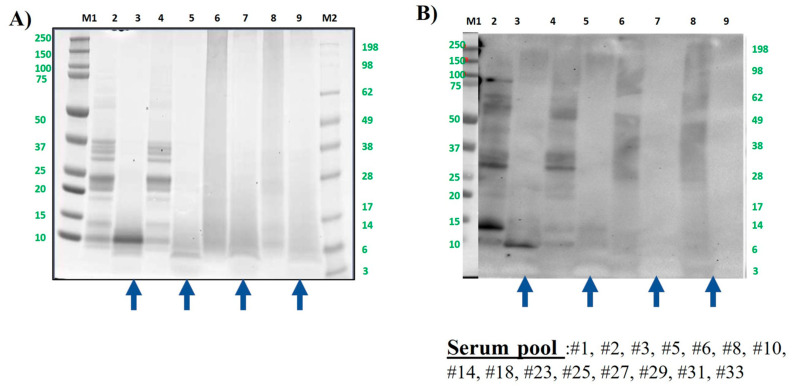
Pistachio protein samples were analyzed by SDS-PAGE electrophoresis in MES 4–12% protein gels (**A**) and by immunoblotting using a pool of sera from pistachio-allergic patients (**B**). Lane 2: control, raw whole pistachio flour (CS). Lane 3: control digested pistachio (DS). Lane 4: boiled for 60 min whole pistachio flour. Lane 5: boiled for 60 min digested pistachio DS. Lane 6: AU 138 °C, 30 min whole pistachio flour. Lane 7: AU 138 °C 30 min digested pistachio DS. Lane 8: DIC 7b, 2 min whole pistachio flour. Lane 9: DIC 7 b, 2 min digested pistachio DS. Lanes M1 and M2: molecular weight markers Precision Plus and See Blue (10–250 kDa, BioRad and 3–198 kDa, Invitrogen, respectively). Bolt LDS sample buffer (Invitrogen) + sample-reducing agent were used in all samples. Digested samples are highlighted with blue arrows under the figure.

The polypeptide profile of pistachio samples, as expected for this nut, ranged between 100 and 5 kDa, being almost the same profile as that of the boiled samples (Figure 4A). The obtained results regarding GI digestion were like those described for hazelnut. After digesting by GI simulation, most of the proteins were eliminated even in the untreated samples (Figure 4A), resulting in only the ones with a molecular weight below 10 kDa, that were indeed immunoreactive by Western blotting (Figure 4B, lane 3). This band, also visible as the typical doublet in lane 2 of raw pistachio pre-digestion, corresponds to the 2S albumin Pis v 1 [15,49].

As observed in previous studies, boiling had no effect on the IgE-binding capacity of pistachio, but after simulated GI digestion, the bands of Pis v 1 were only slightly visible, whereas the immunoreactive bands corresponding to intact and subunits of Pis v 2 (11S albumin, at around 50 kDa and 30, and 20 kDa, respectively) were not present after digestion (lanes 3 and 5 vs. 1 and 4). As confirmed also in previous studies, autoclave and DIC treatment seriously affected the protein and immunogenic patterns of pistachio (lanes 6 and 8 in Figure 4A,B) [32,34].

#### 3.2.4. Effect of Processing on the Cashew Protein Profile and IgE-Binding Capacity After In Vitro Gastrointestinal Digestion

**Figure 5:**
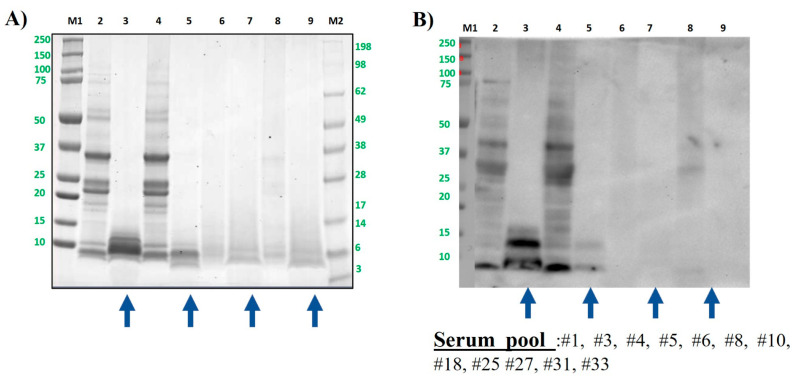
Cashew protein samples were analyzed by SDS-PAGE electrophoresis in MES 4–12% protein gels (**A**) and by immunoblotting using a pool of sera from cashew-allergic patients (**B**). Lane 2: control, raw whole cashew flour (CS). Lane 3: control digested cashew (DS). Lane 4: boiled for 60 min whole cashew flour. Lane 5: boiled for 60 min digested cashew DS. Lane 6: AU 138 °C, 30 min whole cashew flour. Lane 7: AU 138 °C 30 min digested cashew DS. Lane 8: DIC 7b, 2 min whole cashew flour. Lane 9: DIC 7 b, 2 min digested cashew DS. Lanes M1 and M2: molecular weight markers Precision Plus and See Blue (10–250 kDa, BioRad and 3–198 kDa, Invitrogen, respectively). Bolt LDS sample buffer (Invitrogen) + sample-reducing agent were used in all samples. Digested samples are highlighted with blue arrows under the figure.

As seen with pistachio, the cashew protein profile was very similar to that of the untreated and boiled samples, and in general, more reactive than pistachio, in concordance, again, with previous data [32]. After being submitted to GI digestion simulation, protein profiles were slightly different, since in boiled samples, proteins with a lower molecular weight are more retained than in untreated samples (below 10 kDa, Figure 5A, lanes 3 and 5).

That bands or peptide fragments in fact corresponded to Ana o 3 [53], the 2S albumin of cashew, still importantly immunoreactive in untreated nut and significantly less in the boiled sample. Cashew and pistachio shared most of the serum from allergic patients in the assayed pool (Figure S1) and showed a similar capacity to bind IgE before and after the digestion of extremely treated samples. Thus, both autoclave and DIC provoked a strong decrease in the number of immunoreactive bands in the Western blot, as demonstrated previously by our group [31,32,33,34], even though it was possible to visualize some small proteins in SDS-PAGE. In the DIC samples under the assayed conditions, the band at around 30 kDa corresponding to the acidic subunit of Ana o 2, the 11S albumin, was still subtly visible, but after the simulation of GI digestion, no bands were representative in thermally treated cashew.

#### 3.2.5. ELISA Inhibition

**Figure 6:**
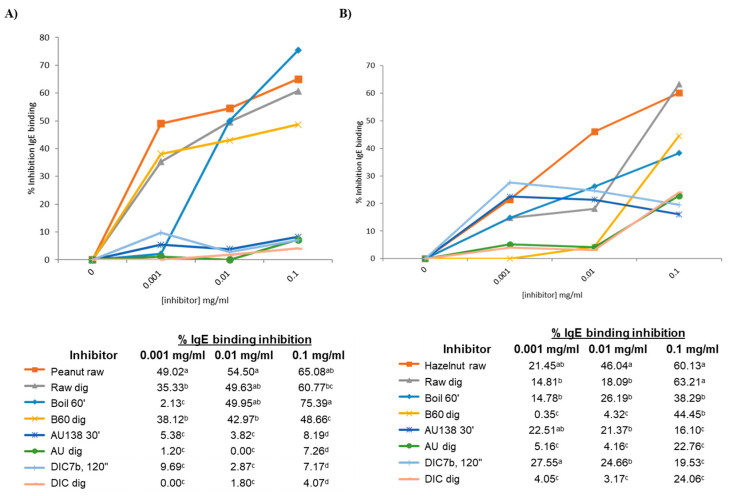
Competitive ELISA inhibition of IgE binding to untreated or raw peanut (**A**) or hazelnut (**B**) by increasing the concentration from 0.001 to 0.1 mg/mL of raw, boiled, autoclaved or DIC-treated samples with and without in vitro digestion. The percentage of inhibition is shown in the table. Significative differences among means are indicated with different superscript letters within the same column, according to Duncan’s test (*p* < 0.05). The color code is indicated in the tables.

A competitive inhibition ELISA was performed using the Sprot pool sera, containing serum from 10 patients reactive to the storage proteins of peanut (Figure S1). Untreated peanut was coated in the plate (control), incubated with inhibitors at different concentrations, and the percentage of inhibition was calculated as described in Section 2.7 in Materials and Methods. Untreated peanut, with and without in vitro digestion, competed for IgE binding to a similar extent at a concentration of 0.1 mg/mL (65.08% and 60.77%) (Figure 6A). In accordance with the immunoblotting, the digestion of boiled peanut had less capacity than the undigested to compete for IgE binding; autoclaved and DIC-treated samples pre- and post-in vitro digestion showed similar results, being the weakest competitors for IgE binding.

Figure 6B shows the capacity of every inhibitor (pre- and post-digestion of untreated and treated hazelnut) to compete with raw samples for binding the IgE from the Sprot pool sera, composed of the serum from seven allergic patients. Again, untreated hazelnut, before and after the INFOGEST protocol, showed a similar percentage of IgE binding inhibition at a maximum concentration of 0.1 mg/mL (60 and 63%), although at lower concentrations, there were significant differences in the competition ability of untreated (raw) samples pre- and post-digestion. Also, at a maximum concentration, digested and non-digested boiled hazelnut showed a similar capacity to bind IgE, whereas by immunoblotting, we observed an important loss of immunoreactive bands in digested samples from untreated and boiled hazelnut. A significant reduction in the percentage of inhibition was in fact observed when the concentration of the inhibitor was lower than 0.1 mg/mL (table below Figure 6B). Interestingly, although by immunoblotting, treated samples by autoclave and DIC were not immunoreactive, we found a range of 16 to 27% of inhibition in the IgE-binding of those samples at every assayed concentration, although that percentage was significantly reduced after digestion with the INFOGEST protocol. Since in ELISA, allergens and proteins are in their native structure, we could assume that some of the allergens may not induce the binding of IgE when proteins or peptides are denatured. Moreover, ELISA is normally more sensitive than Western blotting for detecting proteins in the matrix [52].

Since the behavior of treatments upon GI digestibility, and the protein profiles and IgE-binding capacity resulted were similar among the four assayed nuts, we decided to perform these two inhibition ELISA tests only for peanut and hazelnut, using the Sprot serum pool, as two representative experiments.

## 4. Conclusions

One important characteristic contributing to food allergenicity is resistance to digestion shown by protein allergens. We investigated the stability and IgE-binding capacity of the main allergenic proteins of raw and treated peanut, hazelnut, cashew and pistachio nuts after applying gastric digestion conditions. In this study, we describe that processing based on temperature or temperature and pressure, such as autoclave (thermal) or DIC (non-thermal), affects the extent of the GI digestion of peanut, hazelnut, cashew and pistachio allergens, based on the INFOGEST in vitro protocol. Although GI alone extensively affects protein band profiles, we observed that boiling treatment stimulated GI digestion, whereas drastic treatments with autoclave and DIC themselves were enough to drastically reduce immunoreactivity, even before GI digestion. The application of severe food processing treatments, combined with the study of the GI influence on highly allergenic nuts, might be the first gate for new hypoallergenic formulations. In the future, it would be valuable to use mass spectrometry to characterize residual peptides after gastrointestinal digestion in both raw and processed nuts. Additionally, studying the impact of specific thermal treatments on purified major allergens, and focusing on changes in structure and IgE-binding capacity would be insightful.

## Data Availability

The original contributions presented in the study are included in the article/Supplementary Materials; further inquiries can be directed to the corresponding author.

## Supplementary Materials

The following supporting information can be downloaded at: https://www.mdpi.com/article/10.3390/foods13223549/s1, Figure S1: Distribution of the collected sera by allergenicity to each nut. Table S1: Immunological and clinical information of the 32 patients included in the study.
