# Supplementary material for: Impact of Heat and Pressure Processing Treatments on the Digestibility of Peanut, Hazelnut, Pistachio and Cashew Allergens
Source: Foods. 2024 Nov 7;13(22):3549. doi: 10.3390/foods13223549 (PMC11593142; doi:10.3390/foods13223549)
Supplement: Supplementary file 1 [file foods-13-03549-s001.zip › foods-3284889-supplementary.pdf]

SUPPLEMENTARY MATERIAL

Impact of Heat and Pressure Processing Treatments on Digestibility of Peanut, Hazelnut, Pistachio and Cashew Allergens

Claudia Arribas, Africa Sanchiz, Mercedes M. Pedrosa, Selene Perez, Rosario Linacero and Carmen Cuadrado

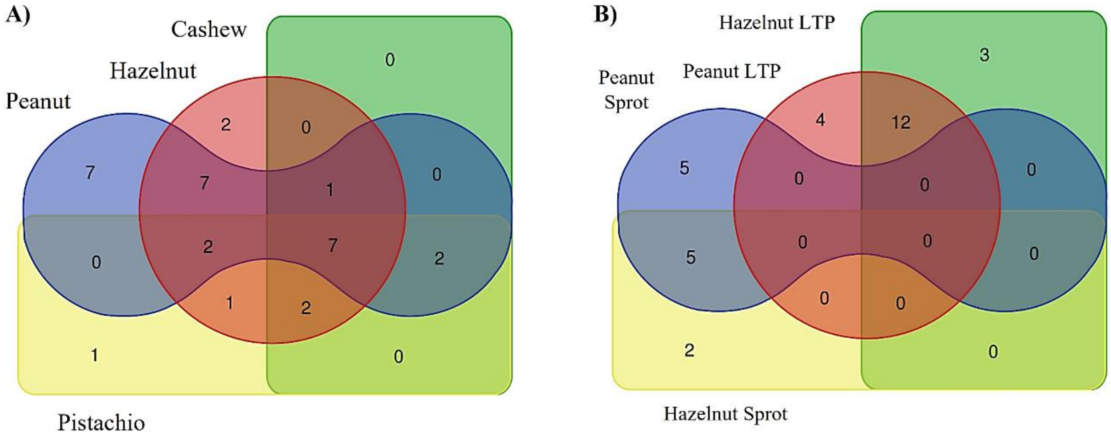

Figure S1. Distribution of collected sera by allergenicity to each nut (**A**) and by kind of allergen in hazelnut and peanut (**B**) (patient #29 excluded)**. Sprot** includes immunoreactivity to storage proteins (2S albumins, 7S vicilins and 11S globulins) from both nuts; LTP refers to those patients who showed reactivity to lipid transfer proteins from both nuts. Venn's diagram were designed with "Bioinformatic and Evolutionary Genomics" webside tool.

Table S1. Immunological and clinical information of the 32 patients included in the study.

Patient no.	Age/Sex	Ig E total (kU/L)	sIg E peanut (kU/L)	sIg E hazelnut (kU/L)	sIg E pistachio (kU/L)	sIg E cashew (kU/L)	Allergen detected											Symptoms	Profile
							Ara h1	Ara h2	Ara h3	Ara h6	Ara h8	Ara h9	Cor a 1	Cor a 8	Cor a 9	Cor a 14	Ana o 3		
# 1	15/M	3291	8.84	28.50	5.50	2.80	--	--	--	--	--	--	--	--	31	--	0.32	OAS	Sprot
# 2	36/F	996	12.10	2.33	1.82	0.57	--	--	--	--	--	3.24	--	0.63	--	--	--	S	LTP
# 3	19/M	5000	16.60	27.90	90.90	53.60	19.5	3.26	0.2	--	--	2.55	--	2.72	63.6	50.8	20.7	S	Sprot
# 4	15/M	2154	14.10	15.90	0.30	0.18	0.1	0.2	0.3	--	--	19.7	--	15		0.16	0.18	S	Sprot/LTP
# 5	19/M	7600	73.60	46.50	1.68	2.79	0.12	70.7	0.24	50.5	--	32.9	--	17.4	3.32	82.7	0.7	S	Sprot/LTP
# 6	17/M	631	6.71	5.27	2.40	1.44	--	--	--	--	--	12.4	--	3.22			1.81	nk	LTP
# 8	16/F	1237	0.58	13.10	1.00	6.35	--	0.61	--	--	--	0.1	Bet v1; 11.7	--	5.16	13.6	0.1	nk	Sprot
# 9	16/F	481	34.10	0.19	0.02	0.18	4.33	12.8	6.36	--	--	--	--	--	--	--	--	nk	Sprot
# 10	52/M	851	0.91	0.37	3.80	3.34	--	--	--	--	--	0.93	--	--	--	--	3.45	--	LTP
# 11	55/F	521	10.50	6.84	2.67	0.08	0.03	0.01	0.02	0.01	0.01	4.3	0.02	3.76	0.5	0.02	0.02	OAS/S	LTP
# 12	51/F	6.8	1.73	0.13	0.01	0.01	--	--	--	--	--	2.77	--	--	--	--	--	OAS	LTP

# 13	55/M	89	1.21	1.23	0.14	0.01	0.01	--	0.02	0.13	--	4.02	0.01	1.28	0.02	0.01	--	OAS/S	LTP
# 14	30/F	453	12.10	4.11	0.46	0.01	0.02	--	0.02	--	0.01	19	0.01	4.34	0.01	0.01	--	S	LTP
# 15	19/F	176	54.90	0.15	0.05	0.03	20.4	24.5	9.05	12.1	--	--	--	--	--	--	--	S	S prot
# 16	23/F	76	2.28	1.91	0.03	0.01	--	--	--	--	0.04	2.28	0.01	1.63		0.01	0.01	OAS	LTP
# 17	39/M	1137	1.46	0.45	0.16	0.03	--	0.62	--	0.06	0.02	1.17	--	--	--	--	--	OAS	Sprot/LTP
# 18	31/F	2211	21.70	11.3	7.0	0.29	0.16	0.12	0.12	0.1	3.46	56.3	13.5	18.3	1.03	0.12	0.13	OAS	LTP
# 19	53/F	49	0.96	2.41	0.19	0	0.01	--	--	--	1.26	1.01	2.82	1.17	0.14	0.01	--	OAS	LTP
#20	25/F	93	0.33	0.38	0.01	0.01	--	--	--	--	--	1.39	--	0.27	--	--	--	OAS	LTP
# 21	6/F	234	>100	0.12	0.53	0.25	2.13	85.5	1.89	53.9	--	--	--	--	--	--		S	Sprot
# 22	29/F	172	0.01	1.72	0.01	0	--	--	--	--	--	--	Bet v1 0.34	--	--	--	--	OAS	Sprot
#23	11/M	2028	0.31	24.0	4.97	6.84	--	--	--	--	--	--		0.46	10.9	0.1	0.15	OAS	LTP
# 24	21/F	110	0.70	3.14	0.36	0	--	--	--	--	--	--	Bet v1 1.73	0.82	0.54	0.13	--	OAS	LTP
# 25	30/M	316	0.40	0.24	0.77	0.97	0.08	0.01	0.01	--	--	0.45	--	--	--	--	0.94	OAS/S	LTP

# 26	36/F	30.4	0.71	1.09	0.02	0	0.01	0.01	0.01	--	0.01	1.14	--	1.15	0.01	0.01	0	OAS	LTP
# 27	14/M	1003	9.76	50.60	26.9	28.1	0.07	7.04	0.06	15	--	--	--	--	45.1	11.9	21.6	nk	Sprot
# 28	23/F	192	0.40	0.18	0.08	0.01	0.01	0.01	0.01	--	0.01	2.99	--	--	--	--		S	LTP
# 29	8/F	102	1.75	3.66	1.81	0.15	--	--	--	--	--	--	Bet v1 4.75	--	0.11	0.04	0.15	--	*PR10*
# 30	34/F	170	2.83	0.33	0.05	0	0.01	0.01	0.01	--	0.01	3.16		1.03		--	0	S	LTP
# 31	11/M	90	0.39	0.02	0.13	0.16	0.74	0.39	--	0.45	--	--	--	--	--	--	0.02	OAS	Sprot
# 32	50/M	150	0.35	0.17	0.15	0.01	0.01	0.01	0.01	--	--	0.13	--	--	--	--		OAS	LTP
# 33	25/F	733	10.5	4.60	0.77	0.13	0.01	0.01	0.01	--	--	17.2	--	1.7	--	--	--	S	LTP

Ax, anaphylaxis; OAS, oral allergy syndrome; S, systemic symptoms (angioderma, urticarial, vomiting…); nk, not know; --, no symptoms.

sIgE, specific IgE; Sprot, storage proteins (2S, 7S o 11S); LTP, ns lipid transfer protein; PR10, pathogenesis related protein 10
